# Reversible NO_2_ Optical Fiber Chemical Sensor Based on LuPc_2_ Using Simultaneous Transmission of UV and Visible Light

**DOI:** 10.3390/s150509870

**Published:** 2015-04-27

**Authors:** Antonio Bueno, Driss Lahem, Christophe Caucheteur, Marc Debliquy

**Affiliations:** 1 Service d’Electromagnétisme et de Télécommunications, Université de Mons, Boulevard Dolez 31, 7000 Mons, Belgium; E-Mail: christophe.caucheteur@umons.ac.be; 2 Materia Nova, Materials R&D Centre, Parc Initialis, Avenue Nicolas Copernic 1, 7000 Mons, Belgium; E-Mail: driss.lahem@materianova.be; 3 Service de Science des Matériaux, Université de Mons, Rue de l’Epargne 56, 7000 Mons, Belgium; E-Mail: marc.debliquy@umons.ac.be

**Keywords:** optical fiber sensor, nitrogen dioxide, lutetium bisphthalocyanine, traffic pollution

## Abstract

In this paper, an NO_2_ optical fiber sensor is presented for pollution monitoring in road traffic applications. This sensor exploits the simultaneous transmission of visible light, as a measurement signal, and UV light, for the recovery of the NO_2_ sensitive materials. The sensor is based on a multimode fiber tip coated with a thin film of lutetium bisphthalocyanine (LuPc_2_). The simultaneous injection of UV light through the fiber is an improvement on the previously developed NO_2_ sensors and allows the simplification of the sensor head, rendering the external UV illumination of the film unnecessary. Coatings of different thicknesses were deposited on the optical fiber tips and the best performance was obtained for a 15 nm deposited thickness, with a sensitivity of 5.02 mV/ppm and a resolution of 0.2 ppb in the range 0–5 ppm. The response and recovery times are not dependent on thickness, meaning that NO_2_ does not diffuse completely in the films.

## 1. Introduction

Nowadays, there is growing concern about toxic gases and air contaminants due to the increasing consumption of fossil fuels. Indeed, the combustion of these fuels leads to a massive release of nitrogen oxides (NO_x_) and other pollutants, such as SO_2_, CO and CO_2_. This can cause environmental issues such as the greenhouse effect, acid rain, production of ozone in low atmosphere and air pollution (known as “smog”) [1]. In recent decades, the increase of road traffic has made the emissions of internal combustion engines the main source of NO_x_, which is one of the main toxic gases that can cause respiratory and coronary diseases [2]. For this reason, the need for gas sensors suitable for monitoring and controlling NO_2_ has arisen. Numerous efforts have been made in the development of NO_2_ gas sensors.

Among the different technologies, optical fiber based systems can be very useful in specific applications like the monitoring of road tunnels. The techniques already employed are all based on the deposition of a sensitive layer reacting with the gases, which change the optical properties of this layer (complex refractive index). Previously reported works used the deposition of a sol gel coating on a fiber core [3], on a fiber with a reduced diameter [4], on fiber Bragg gratings [5,6] or simply on the tip of a fiber [7,8]. Concerning the optical fiber sensors already developed for NO_2_ measurement, one of the first configurations used sections of chemically pretreated porous silica fiber [9], which presented changes to the transmission spectra under NO_2_ exposure. In [10], the sensor element consisted of the replacement of a portion of the cladding region of a multimode plastic clad silica fiber by metallophthalocyanines such as CuPc, PbPc and SmPc. There are some other works where the sensing element is extrinsic to the optical fiber. This is because the optical fiber is only used to transmit/receive the optical signal after the reflection in the sensitive material deposited in a sol-gel film [11] or a disc [12] or after transmission passing through a sensing plate [13].

In this work, the results obtained with an NO_2_ optical fiber sensor based on a coated fiber tip, using LuPc_2_ as a sensitive molecule, are presented. The sensitive film experiences a variation of reflectance due to the decreased absorption at 660 nm following NO_2_ adsorption. The adsorption is reversible but very slow at room temperature. So as to shorten the recovery time [12], the films were exposed to ultraviolet (UV) light at 365 nm. In order to get a practical system, both UV and red lights were injected into the same fiber, avoiding the need for an external UV source, and achieving a high power density of UV with a low power source thanks to the reduced size of the fiber core. This feature allows the simplification of the sensor head, making the external UV illumination of the film, reported in the works published up to now, unnecessary.

## 2. Material and Methods

### 2.1. Preparation of the Sensors

**Figure 1:**
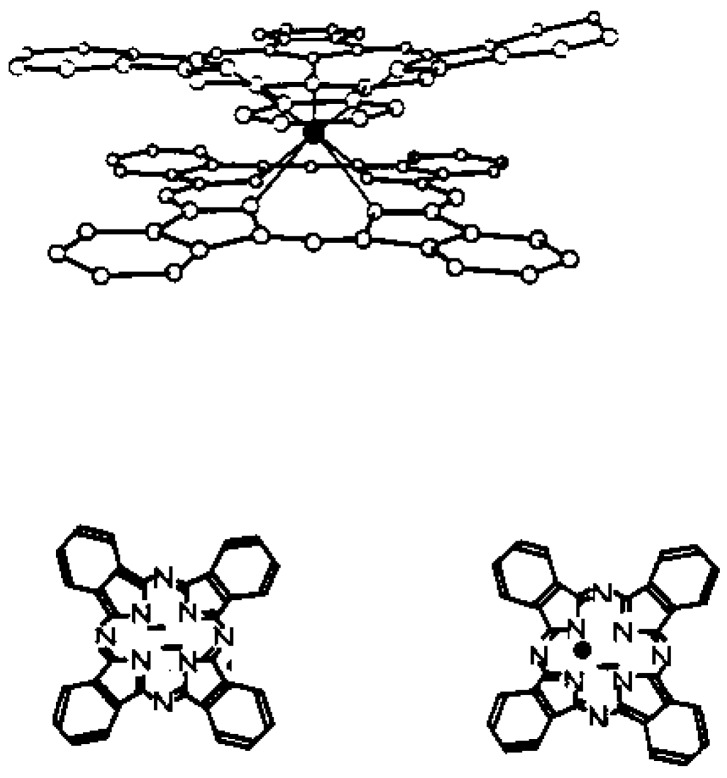
Molecular structure of lutetium bisphthalocyanine (LuPc_2_).

Phthalocyanines are good materials for gas sensing purposes due to their electrochemical and optical properties [14,15,16]. These organic molecules are known to be sensitive to oxidizing or reducing gases at ppm concentrations. For this reason they have been proposed as the chemically active component of both conductive and optical gas sensors [17,18,19]. Another major advantage is the remarkable chemical and thermal stability of the phthalocyanine derivatives in many environmental conditions. Lanthanide bisphthalocyanine (LnPc_2_) complexes with a “double-decker” structure [20], like that presented in Figure 1 for LuPc_2_, are a typical class of compound with π-π* transitions and they exist in different forms associated with different colors. The green species is a neutral form, the red form is a singly oxidized species and the blue form is a singly reduced species. The study of lanthanide diphthalocyanines is very attractive due to their electrochromic properties [21,22,23] and semiconducting behaviors [24]. Among these lanthanide diphthalocyanines, the bisphtalocyanine of lutetium LuPc_2_ was chosen as the model compound for application in the present study.

Low ionization energy together with high polarization energy makes the transfer of charge between LuPc_2_ and acceptor molecules easier. The transfer of electrons impacts the absorption spectra or the conductivity of LuPc_2_ in the UV, visible and near infrared ranges. These molecules were studied for gas detection [25,26,27].

The interaction of the LuPc_2_ molecules in the presence of NO_2_ gas is described in Equation (1). This reaction is reversible.

LuPc_2_ + NO_2_ ↔ LuPc_2_^+^ + NO_2_^−^ (1)

The sensor consists of a thin layer of LuPc_2_, deposited by evaporation on the tip of an optical fiber. The optical fiber is a pure silica 400 µm core diameter multimode fiber covered with a 12.5 µm thickness hard polymer as cladding. This optical fiber has a high OH content in order to reduce the optical transmission losses at UV and visible wavelengths.

Lutetium bisphthalocyanine (LuPc_2_) was synthetized by Bouvet (University of Burgundy, France) from the o-dicyanobenzene by heating it with lutetium triacetate, Lu(OAc)_3_, at approximately 300 °C, without any solvent according to [28].

The LuPc_2_ thin films were deposited in a BOC Edwards Auto 306 Evaporator by thermal sublimation at high vacuum (p = 2 × 10^−6^ mbar) with thicknesses ranging from 15 to 90 nm. The substrate was kept at room temperature. The deposition rate was ~5 nm/min and the film thickness was controlled *in situ* by using a quartz crystal thickness monitor located in the deposition chamber.

The LuPc_2_ deposits were carried out on the multimode optical fibers and on flat glass substrates in order to characterize the response to NO_2_ exposure on large samples.

### 2.2. Effect of NO_2_ on the Absorption Spectrum of LuPc_2_ and Accelerated Recovery by UV Light Illumination

**Figure 2:**
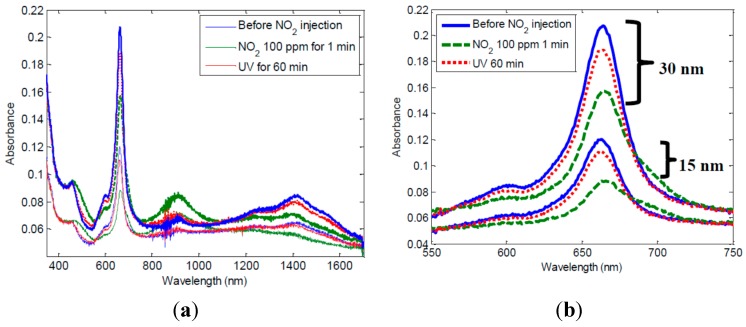
Absorbance spectra of pure LuPc_2_ thin films (**a**) whole spectra; (**b**) zoom on 660 nm.

Figure 2a,b show the absorbance spectra for 15 nm and 30 nm thicknesses of LuPc_2_ films on glass substrate, before and after contact with NO_2_. The film is originally green and fades to a reddish color after NO_2_ exposure. The spectrum is affected in the wavelength range between 400 nm and 1600 nm. Several absorption bands can be chosen to monitor optical changes. The most intense absorption band (Q band) at around 660 mm was chosen for this study. It can be seen that the strong absorption peak intensity decreases after only 1 min of NO_2_ exposure at a concentration of 100 ppm. This effect is reversible, but more than 24 h are needed at room temperature to recover the original spectrum. This is due to the high stability of the (LuPc_2_ + NO_2_^−^) complex formed between NO_2_ and LuPc_2_, which induces a slow desorption of NO_2_.

However, it can be observed that UV illumination accelerates the recovery (the absorption peak increases after UV exposure) as shown by Baldini *et al* [12]. For this test, samples were placed in a 10 mW/cm^2^ UV furnace at 365 nm for 60 min. This accelerated recovery is related to photodissociation of NO_2_ under UV light. The NO_2_ photodissociation under UV illumination at wavelengths below 420 nm can be expressed as:

NO_2_ + hν → NO + O (2)

The photodissociation rate for NO_2_ molecules under UV light illumination is proportional to the NO_2_ absorption cross-section and the quantum-yield for photodissociation [29], having a maximum rate in the range 360–395 nm. For the present experiments, a commercial LED source at 365 nm was used. It can be assured that prolonged exposures of the films at this wavelength do not destroy the films.

### 2.3. Measurement Setup

**Figure 3:**
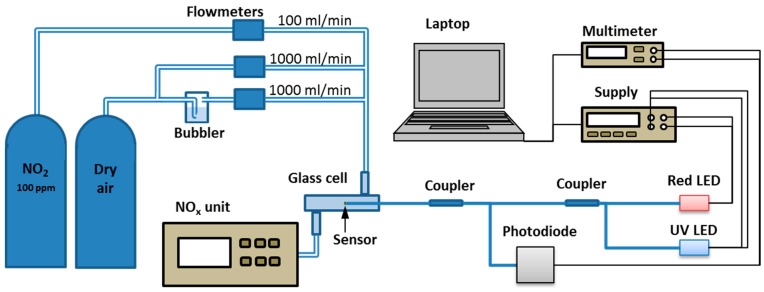
Experimental setup for NO_2_ measurements.

The experimental setup for NO_2_ measurement is shown in Figure 3. In the gas control part, a compressed air bottle was hooked up to two flow meters in order to control the relative humidity by changing the dry air/wet air relationship. A third flowmeter was connected to a 100 ppm NO_2_ bottle. The outputs from the three flowmeters were interconnected and attached to a glass cell where the optical fiber sensors were placed. In the output of the glass cell a NO_x_ chemiluminescence analyzer was connected in order to measure the NO_2_ concentration in the cell.

In the optical part, two LEDs were used: a red one (emitting at 660 nm) and a UV one (emitting at 365 nm). A 200 µm core multimode optical fiber was connected to the UV LED, obtaining 0.5 mW of optical output power. A 400 µm core multimode optical fiber was connected to the red LED, yielding an optical output power of 14.5 mW. Both LEDs were connected to an optical coupler with a 50:50 ratio in order to combine the two wavelengths in the same fiber. The output of the coupler was connected to the input of an identical coupler. At the output of the second coupler a 400 µm core multimode fiber was connected, having the sensitive coating on the fiber end. Light was reflected by the fiber end, heading back to the optical coupler, and it was finally detected by a photodiode. The optical signal was converted into an electrical signal and it was measured with a multimeter. Because UV light disturbs the measurement by adding a superimposed voltage level, a laptop controlled the source to be synchronized with the multimeter. In order to take a measurement, the power supply of the UV LED was disabled and the voltage measured by the multimeter was registered. After that, the UV LED was switched on again. The power density at the end of the fiber was about 290 W/m^2^.

## 3. Results and Discussion

### 3.1. Response of the Optical Fiber Sensor 

**Figure 4:**
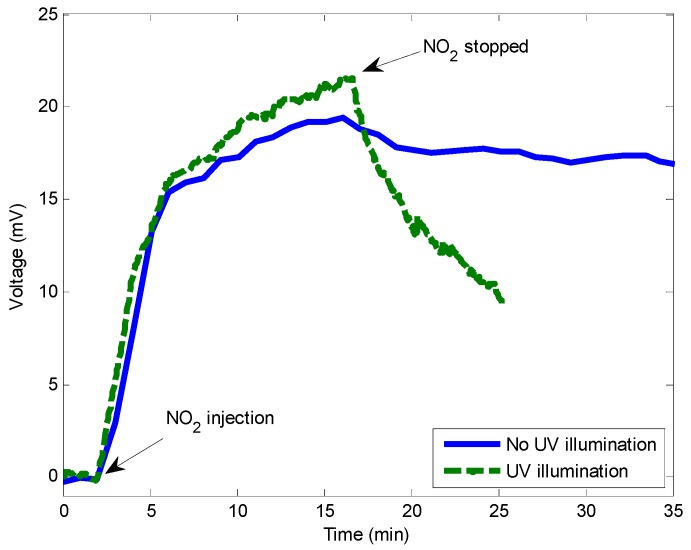
Response of a 15 nm thickness sensor without and with UV light illumination.

The test presented in Figure 4 consisted of applying a flow of 4 ppm NO_2_ for 15 min in humid air (50% RH) and after that, stopping the flow of NO_2_ without applying UV illumination. As can be seen in Figure 4, the sensor reacted to the presence of NO_2_ leading to an increase of the signal corresponding to an increasing reflectance of the sample. When the flow of NO_2_ was stopped, the signal decreased very slowly since NO_2_ desorption is very slow. The same experiment was then conducted with a simultaneous injection of UV light into the fiber. The adopted UV light illumination strategy was to activate the UV LED continuously, but since UV light disturbs the photodiode adding a superimposed voltage level, the UV LED was disabled every 5 s in order to register the voltage for 1 s. Figure 4 shows that the use of UV-light leads to a drastic reduction of the recovery time.

### 3.2. Effect of the Thickness 

Up to three different coating thicknesses were deposited on 400 µm core multimode fiber tips: 15 nm, 30 nm and 45 nm. Optical sensors were placed inside the glass cell and a relative humidity of 50% was applied. Experiments started 1 h after signal stabilization.

**Figure 5:**
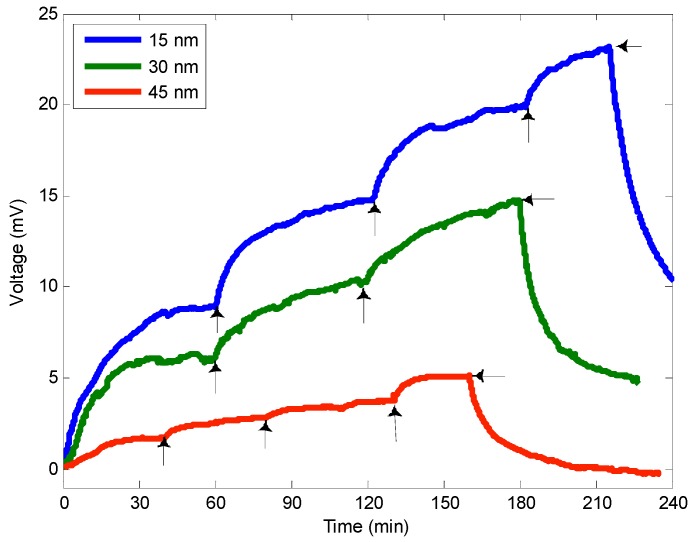
Response of the optical fiber sensors to different NO_2_ concentrations.

Sensor responses to different NO_2_ concentrations were measured. The NO_2_ concentration applied to the optical sensors was increased in steps (25%, 50%, 75% and 100% of the NO_2_ flowmeter capacity). These flow values correspond to measured NO_2_ concentration values of 0.95 ppm, 2.00 ppm, 3.05 ppm and 4.10 ppm, respectively. The signal evolution from the different optical fiber sensors is shown in Figure 5. Sensors react to the increasing NO_2_ concentrations by an increase of the signal on the photodiode. The injection times of NO_2_ were not the same for the different sensors. These are marked in Figure 5 with arrows.

**Figure 6:**
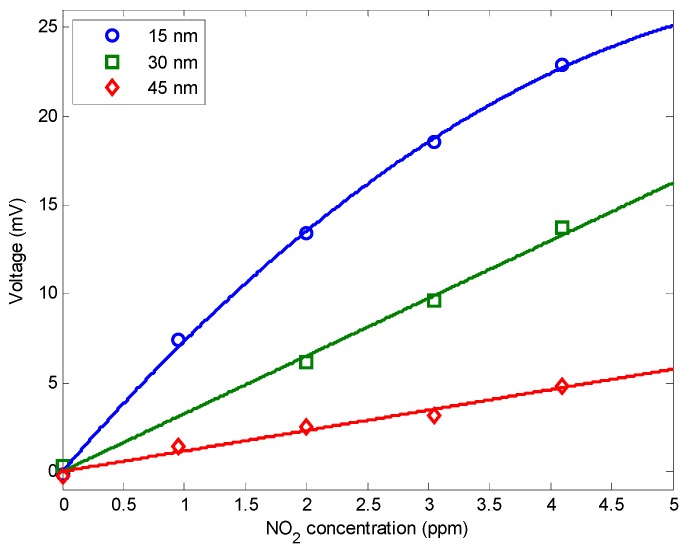
Calibration of the optical fiber sensors with NO_2_ concentration.

Figure 6 depicts the response after stabilization versus NO_2_ concentration for different thicknesses. The signal decreases when the thickness increases, which means that the reaction remains close to the film surface. Moreover, the response time was not dramatically increased by an increase of the thickness (see Table 1) confirming that gas does not diffuse throughout the film. If this were the case, the response time would be multiplied by a factor four when doubling the thickness according to the classical Fick diffusion equations.

The response of the 15 nm thickness sensor shows a non-linear response, probably due to a saturation mechanism, whereas the 30 nm and the 45 nm can be linearly fitted. The sensitivities of the sensors with coating thicknesses of 15 nm, 30 nm and 45 nm can be calculated as S_15nm_ = 5.02 mV/ppm, S_30nm_ = 3.25 mV/ppm and S_45nm_ = 1.15 mV/ppm, respectively. Using a digital multimeter with a display resolution of 6½ digits, a voltage resolution of 0.001 mV can be obtained, corresponding to an NO_2_ concentration resolution of 0.2 ppb, 0.3 ppb and 0.9 ppb for thicknesses of 15 nm, 30 nm and 45 nm, respectively.

### 3.3. Repeatability

**Figure 7:**
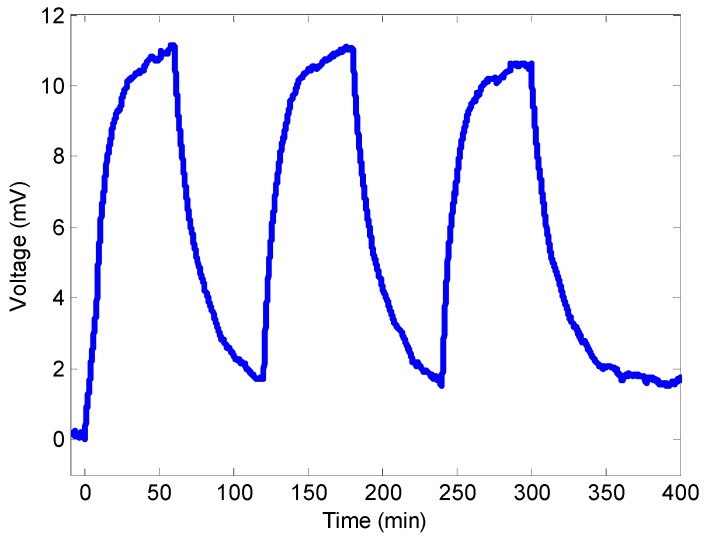
Repeatability performance for the 15 nm thickness NO_2_ optical fiber sensor.

Repeatability tests were also carried out. The response of the 15 nm thickness sensor to consecutive injections of 2 ppm NO_2_ is depicted in Figure 7. As it can be seen, the response is quite repeatable. The standard deviation can be calculated as 5.54%. The repeatability performances for the 30 nm and 45 nm thicknesses sensors are similar. Concerning the 15 nm thickness sensor, the mean response time (defined as the time needed to reach the 90% of the final voltage) was 25 min and the mean recovery time (defined as the time needed to reach the 10% of the final voltage) was 38 min. Concerning the 30 nm thickness sensor, the response time was 27 min and the recovery time was 35 min. Finally, for the 45 nm thickness sensor, the response time was 27 min and the recovery time was 40 min. The response or recovery time does not drastically depend on the thickness, confirming that NO_2_ does not diffuse completely in the film.

To summarize, the relationship between the detected voltage and the NO_2_ concentration is given in Table 1.

**Table 1:** Characterization of the sensors depending on their thicknesses.

	Sensibility	Resolution	Response time	Recovery Time
15 nm	5.02 mV/ppm	0.2 ppb	25 min	38 min
30 nm	3.25 mV/ppm	0.3 ppb	27 min	35 min
45 nm	1.15 mV/ppm	0.9 ppb	27 min	40 min

Compared to a previously developed NO_2_ optical fiber sensors [12] working in a similar range of NO_2_ concentrations (between 0 ppm and 10 ppm), the sensor presented in this article has lower response and recovery times, but most importantly, the UV illumination for signal recovery can be done without direct intervention. UV light can be transmitted through the fiber and there is no need to disconnect the fiber in order to illuminate the sensitive material with an external UV lamp. In a more recent work [13], the sensor presented worked in a higher NO_2_ concentration range (between 0 ppm and 30 ppm), with a similar response and recovery time. Nevertheless, the recovery time can be decreased, but only by applying heat and a vacuum to the sensitive material. It has to be noted that the two aforementioned sensors are extrinsic to the optical fiber, since the sensitive material is coated in an external support (a membrane or a plate), making the sensor head more complex and bigger in size.

### 3.4. Sensor Modeling

In this section, the equations describing the behavior of the optical fiber NO_2_ sensor are developed. The measurand related to the environment (NO_2_ concentration) in the setup presented in Figure 3 is the photodetector voltage as a result of the photodetected optical power reflected from the sensor. The photodetector voltage can be calculated with Equation (3):

(3) $${\text{V}}_{\text{PD}}={\text{P}}_{\text{in}}\cdot {ℜ}_{\text{PD}}\cdot {\text{G}}_{\text{PD}}$$

where P_in_ is the input power to the photodetector in W, ℜ_PD_ is the responsivity of the photodiode in A/W, and G_PD_ is the transimpedance gain of the photodiode in V/A. The input power to the photodiode can be calculated with following equation:

(4) $$P_{in}=\frac{P_{LED}}{L_{T}}\cdot R=P_{LED}\cdot G_{T}\cdot R$$

where P_LED_ is the output power of the red LED (emitting at 660 nm) in W, L_T_ is the total power loss of the optical power in the optical setup (including connector losses and power splitting and coupling efficiency in the optical couplers) and R is the combined reflectance of the fiber and the sensitive layer. The optical losses can be considered as a gain as the G_T_ value is lower than 1. By incorporating Equation (4) into Equation (3), it can be seen that the photodetector voltage depends on the reflectance, since the other parameters are constant:

(5) $$V_{PD}=P_{LED}\cdot G_{T}\cdot {ℜ}_{PD}\cdot G_{PD}\cdot R=K\cdot R$$

The reflectance of the combination of the fiber and sensitive layer can be described as the sum of the reflectance in the fiber-layer interface (R_fl_) and the reflectance in the layer-air interface (R_la_):

(6) $$R\approx R_{fl}+R_{la}\cdot e^{-2\alpha d}$$

**Figure 8:**
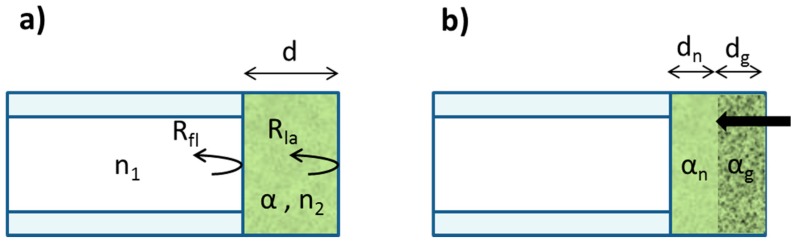
Sensor representation (**a**) before and (**b**) after NO_2_ exposure.

The light reflected in the layer-air interface experiences attenuation due to the absorption of the sensitive layer of length d, characterized by an absorption coefficient α, expressed in m^−1^. This coefficient is homogeneous in the sensitive layer but after NO_2_ exposure there is a diffusion of the gas through the layer, decreasing the absorbance (as seen in Figure 2) in a length d_g_, as is depicted in Figure 8b. Thus, after NO_2_ exposure the total absorption in the layer can be described as follows:

(7) $$\alpha d=\alpha_{n}d_{n}+\alpha_{g}d_{g}$$

where α_n_ is the absorption coefficient of the sensitive layer in a normal situation (before NO_2_ exposure) in the unaffected length d_n_ and α_g_ is the absorption coefficient in the length d_g_ where the NO_2_ gas penetrates.

Inserting Equations (7) and (6) into Equation (5), the photodetector voltage can be expressed as:

$$V_{PD}=K\left[R_{fl}+R_{la}\cdot e^{-2\alpha d}\right]=K\left[R_{fl}+R_{la}\cdot e^{-2\left(\alpha_{n}d_{n}+\alpha_{g}d_{g}\right)}\right]=K\left[R_{fl}+R_{la}\cdot e^{-2\left(\alpha_{n}\left(d-d_{g}\right)+\alpha_{g}d_{g}\right)}\right]=K\left[R_{fl}+R_{la}\cdot e^{-2\alpha_{n}d}\cdot e^{-2\left(\alpha_{g}-\alpha_{n}\right)d_{g}}\right]\approx K\left[R_{fl}+R_{la}\cdot e^{-2\alpha_{n}d}\left(1+2\left(\alpha_{n}-\alpha_{g}\right)d_{g}\right)\right]$$

(8) $$V_{PD}=K\left[R_{fl}+R_{la}\cdot e^{-2\alpha_{n}d}\right]+2K\left[R_{la}\cdot e^{-2\alpha_{n}d}\left(\alpha_{n}-\alpha_{g}\right)d_{g}\right]=V_{0}+\Delta V_{PD}$$

In Equation (8), the first term V_0_ is a baseline and the voltage variations are encoded in the second term.

(9) $$\Delta V_{PD}=2KR_{la}\cdot e^{-2\alpha_{n}d}\left(\alpha_{n}-\alpha_{g}\right)d_{g}$$

The variation of NO_2_ concentration modifies the parameters α_g_ and d_g_ since the penetration of the NO_2_ molecules and the absorption depend on the thickness of the layer and the NO_2_ concentration as can be seen in Figure 2. According to Equation (9), and due to the fact that the gas does not diffuse in the whole layer, the voltage variation ΔV_PD_ depends on the thickness of the layer. When d increases the sensitivity decreases, as was experimentally observed.

## 4. Conclusions

An optical fiber chemical sensor based on LuPc_2_ thin films has been developed for NO_2_ detection, which is useful for the monitoring of traffic pollution. A special fiber was selected in order to allow the transmission of both UV and visible light through the fiber, improving the previously developed NO_2_ sensors by the simplification of the sensor head, since there is no need to use an external UV source to illuminate the films.

The sensitive coating was deposited by thermal evaporation onto the fiber tip. Absorption at 660 nm was monitored. Contact with NO_2_ drastically and reversibly decreased absorption. Due to a strong interaction with NO_2_, the LuPc_2_ thin films showed good sensitivity for low concentrations, but the recovery time was very long. To reduce the recovery time, the films were irradiated by UV light at 365 nm in order to promote the photodissociation of the complex formed between NO_2_ and LuPc_2_ molecules.

In this study, the sensors demonstrated the possibility of monitoring NO_2_ concentration in the range 0–5 ppm. Sensitivity decreases when the film thickness increases, but the impact of the thickness on the response or recovery time is less important. This means that the reaction essentially takes place close to the surface. A higher sensitivity of 5.02 mV/ppm was obtained with the 15 nm coating thickness sensor, also achieving lower response and recovery times of 25 min and 38 min, respectively. A resolution of 0.2 ppb was obtained with this sensor.

## References

[1] Olivier J.J.M., Bouwman J.G.J., van der Hoek A.F., Berdowski K.W. (1998). Global air emission inventories for anthropogenic sources of NO_x_, NH_3_ and N_2_O in 1990. Environ. Pollut..

[2] Maître A., Bonneterre V., Huillard L., Sabatier P., Gaudemaris R. (2006). Impact of urban atmospheric pollution on coronary disease. Eur. Heart J..

[3] Mac Craith B.D., O’Keefe G., McDonagh C., McEvoy A.K. (1994). LED-based fibre optic oxygen sensor using sol-gel coating. Electron. Lett..

[4] Bariáin C., Matías I.R., Arregui F.J., López-Amo M. (1998). Experimental results towards development of humidity sensors by using hygroscopic material on biconically tapered optical fiber. Proc. SPIE.

[5] Caucheteur C., Debliquy M., Lahem D., Mégret P. (2008). Catalytic fiber Bragg grating sensor for hydrogen leak detection in air. IEEE Photon. Technol. Lett..

[6] Caucheteur C., Debliquy M., Lahem D., Mégret P. (2008). Hybrid fiber gratings coated with a catalytic sensitive layer for hydrogen sensing in air. Opt. Express.

[7] Mechery S.J., Singh J.P. (2006). Fiber optic based gas sensor with nanoporous structure for the selective detection of NO_2_ in air samples. Anal. Chimica Acta.

[8] Bezunartea M., Estella J., Echeverría J.C., Elosúa C., Bariáin C., Laguna M., Luquin A., Garrido J.J. (2008). Optical fibre sensing element based on xerogel-supported [Au_2_Ag_2_(C_6_F_5_)_4_(C_14_H_10_)]n for the detection of methanol and ethanol in the vapour phase. Sens. Actuators B Chem..

[9] Schmidlin E.M., Mendoza E.A., Ferrell D.J., Syracuse S.J., Khalil A.N., Lieberman R.A. (1994). A fiber optic NO_2_ sensor for combustion monitoring. Proc. SPIE.

[10] John M.S., Unnikrishnan K.P., Thomas J., Radhakrishnan P., Nampori V.P.N., Vallabhan C.P.G. Characterization of an optical fiber sensor in detecting NO_2_ gas. Proceedings of the International Conference on Fiber Optics & Photonics PHOTONICS-2000.

[11] Mechery S.J., Singh J.P. (2004). Self-calibrated fiber optic transflection probe for NO_2_ detection. Proc. SPIE.

[12] Baldini F., Capobianchi A., Falai A., Mencaglia A.A., Pennesi G. (2001). Reversible and selective detection of NO_2_ by means of optical fiber. Sens. Actuators B Chem..

[13] Ohira S.-I., Wanigasekara E., Rudkevich D.M., Dasgupta P.K. (2009). Sensing parts per million levels of gaseous NO_2_ by an optical fiber transducer based on calix[4]arenes. Talanta.

[14] Leznoff C.C., Lever A.B.P. (1989). Phthalocyanines: Properties and Applications.

[15] McKeown N.B. (1998). Phthalocyanine Materials: Synthesis, Structure and Function. Chemistry of Solid State Materials.

[16] Simon J., André J.-J. (1985). Molecular Semiconductors.

[17] Wright J.D. (1991). Gas adsorption on phthalocyanines and its effects on electrical properties. Prog. Surf. Sci..

[18] Mukhopadhyay S., Hogarth C.A. (1994). Gas sensing properties of phthalocyanine Langmuir–Blodgett films. Adv. Mater..

[19] Capone S., Mongelli S., Rella R., Siciliano P., Valli L. (1999). Gas sensitivity measurements on NO_2_ sensors based on Coper(II) tetrakis(n-butylaminocarbonyl) phthalocyanine LB films. Langmuir.

[20] Simon J., Bouvet M., Bassoul P. (1994). The Encyclopedia of Advanced Materials.

[21] Rodríguez-Méndez M.L., Gorbunova Y., de Saja J.A. (2002). Spectroscopic Properties of Langmuir−Blodgett Films of Lanthanide Bis(phthalocyanine)s Exposed to Volatile Organic Compounds. Sensing Applications. Langmuir.

[22] Rodriguez-Mendez M.L., Aroca R., DeSaja J.A. (1993). Electrochromic and gas adsorption properties of Langmuir-Blodgett films of lutetium bisphthalocyanine complexes. Chem. Mater..

[23] Rodriguez-Mendez M.L., Aroca R., DeSaja J.A. (1992). Electrochromic properties of Langmuir-Blodgett films of bisphthalocyanine complexes of rare earth elements. Chem. Mater..

[24] Maitrot M., Guillaud G., Boudjema B., André J.-J., Strzelecka H., Simon J., Even R. (1987). Lutetium bisphthalocyanine: The first molecular semiconductor. Conduction properties of thin films of p- and n-doped materials. Chem. Phys. Lett..

[25] Gutierrez N., Rodríguez-Méndez M.L., de Saja J.A. (2001). Array of sensors based on lanthanide bisphtahlocyanine Langmuir–Blodgett films for the detection of olive oil aroma. Sens. Actuators B Chem..

[26] De Saja J.A., Rodríguez-Méndez M.L. (2005). Sensors based on double-decker rare earth phthalocyanines. Adv. Colloid Interf. Sci..

[27] Bariáin C., Matías I.R., Fernández-Valdivielso C., Arregui F.J., Rodríguez-Méndez M.L., de Saja J.A. (2003). Optical fiber sensor based on lutetium bisphthalocyanine for the detection of gases using standard telecommunication wavelengths. Sens. Actuators B Chem..

[28] Clarisse C., Riou M.-T. (1987). Synthesis and characterization of some lanthanide phthalocyanines. Inorg. Chimica Acta.

[29] Parrish D.D., Murphy P.C., Albritton D.L., Fehsenfeld F.C. (1983). The measurement of the photodissociation rate of NO_2_ in the atmosphere. Atmos. Environ..

